# CRdb: a comprehensive resource for deciphering chromatin regulators in human

**DOI:** 10.1093/nar/gkac960

**Published:** 2022-11-01

**Authors:** Yimeng Zhang, Yuexin Zhang, Chao Song, Xilong Zhao, Bo Ai, Yuezhu Wang, Liwei Zhou, Jiang Zhu, Chenchen Feng, Liyan Xu, Qiuyu Wang, Hong Sun, Qiaoli Fang, Xiaozheng Xu, Enmin Li, Chunquan Li

**Affiliations:** The Key Laboratory of Molecular Biology for High Cancer Incidence Coastal Chaoshan Area, Shantou University Medical College, Shantou 515041, China; The First Affiliated Hospital, Institute of Cardiovascular Disease, Hengyang Medical School, University of South China, Hengyang, Hunan 421001, China; School of Computer, University of South China, Hengyang, Hunan 421001, China; The First Affiliated Hospital, Cardiovascular Lab of Big Data and Imaging Artificial Intelligence, Hengyang Medical School, University of South China, Hengyang, Hunan 421001, China; Hunan Provincial Base for Scientific and Technological Innovation Cooperation, University of South China, Hengyang, Hunan 421001, China; The First Affiliated Hospital, Department of Cardiology, Hengyang Medical School, University of South China, Hengyang, Hunan 421001, China; The First Affiliated Hospital, Institute of Cardiovascular Disease, Hengyang Medical School, University of South China, Hengyang, Hunan 421001, China; School of Computer, University of South China, Hengyang, Hunan 421001, China; The First Affiliated Hospital, Cardiovascular Lab of Big Data and Imaging Artificial Intelligence, Hengyang Medical School, University of South China, Hengyang, Hunan 421001, China; Hunan Provincial Base for Scientific and Technological Innovation Cooperation, University of South China, Hengyang, Hunan 421001, China; The First Affiliated Hospital, Department of Cardiology, Hengyang Medical School, University of South China, Hengyang, Hunan 421001, China; School of Medical Informatics, Daqing Campus, Harbin Medical University. Daqing 163319, China; School of Medical Informatics, Daqing Campus, Harbin Medical University. Daqing 163319, China; School of Medical Informatics, Daqing Campus, Harbin Medical University. Daqing 163319, China; School of Medical Informatics, Daqing Campus, Harbin Medical University. Daqing 163319, China; School of Medical Informatics, Daqing Campus, Harbin Medical University. Daqing 163319, China; School of Medical Informatics, Daqing Campus, Harbin Medical University. Daqing 163319, China; The Key Laboratory of Molecular Biology for High Cancer Incidence Coastal Chaoshan Area, Shantou University Medical College, Shantou 515041, China; Guangdong Provincial Key Laboratory of Infectious Diseases and Molecular Immunopathology, Institute of Oncologic Pathology, Cancer Research Center, Shantou University Medical College, Shantou 515041, China; The First Affiliated Hospital, Institute of Cardiovascular Disease, Hengyang Medical School, University of South China, Hengyang, Hunan 421001, China; School of Computer, University of South China, Hengyang, Hunan 421001, China; The First Affiliated Hospital, Cardiovascular Lab of Big Data and Imaging Artificial Intelligence, Hengyang Medical School, University of South China, Hengyang, Hunan 421001, China; Hunan Provincial Base for Scientific and Technological Innovation Cooperation, University of South China, Hengyang, Hunan 421001, China; The First Affiliated Hospital, Department of Cardiology, Hengyang Medical School, University of South China, Hengyang, Hunan 421001, China; The Key Laboratory of Molecular Biology for High Cancer Incidence Coastal Chaoshan Area, Shantou University Medical College, Shantou 515041, China; Department of Biochemistry and Molecular Biology, Shantou University Medical College, Shantou 515041, China; The First Affiliated Hospital, Institute of Cardiovascular Disease, Hengyang Medical School, University of South China, Hengyang, Hunan 421001, China; School of Computer, University of South China, Hengyang, Hunan 421001, China; School of Medical Informatics, Daqing Campus, Harbin Medical University. Daqing 163319, China; The Key Laboratory of Molecular Biology for High Cancer Incidence Coastal Chaoshan Area, Shantou University Medical College, Shantou 515041, China; Department of Biochemistry and Molecular Biology, Shantou University Medical College, Shantou 515041, China; The First Affiliated Hospital, Institute of Cardiovascular Disease, Hengyang Medical School, University of South China, Hengyang, Hunan 421001, China; School of Computer, University of South China, Hengyang, Hunan 421001, China; The First Affiliated Hospital, Cardiovascular Lab of Big Data and Imaging Artificial Intelligence, Hengyang Medical School, University of South China, Hengyang, Hunan 421001, China; Hunan Provincial Base for Scientific and Technological Innovation Cooperation, University of South China, Hengyang, Hunan 421001, China; The First Affiliated Hospital, Department of Cardiology, Hengyang Medical School, University of South China, Hengyang, Hunan 421001, China

## Abstract

Chromatin regulators (CRs) regulate epigenetic patterns on a partial or global scale, playing a critical role in affecting multi-target gene expression. As chromatin immunoprecipitation sequencing (ChIP-seq) data associated with CRs are rapidly accumulating, a comprehensive resource of CRs needs to be built urgently for collecting, integrating, and processing these data, which can provide abundant annotated information on CR upstream and downstream regulatory analyses as well as CR-related analysis functions. This study established an integrative CR resource, named CRdb (http://cr.liclab.net/crdb/), with the aim of curating a large number of available resources for CRs and providing extensive annotations and analyses of CRs to help biological researchers clarify the regulation mechanism and function of CRs. The CRdb database comprised a total of 647 CRs and 2,591 ChIP-seq samples from more than 300 human tissues and cell types. These samples have been manually curated from NCBI GEO/SRA and ENCODE. Importantly, CRdb provided the abundant and detailed genetic annotations in CR-binding regions based on ChIP-seq. Furthermore, CRdb supported various functional annotations and upstream regulatory information on CRs. In particular, it embedded four types of CR regulatory analyses: CR gene set enrichment, CR-binding genomic region annotation, CR-TF co-occupancy analysis, and CR regulatory axis analysis. CRdb is a useful and powerful resource that can help in exploring the potential functions of CRs and their regulatory mechanism in diseases and biological processes.

## INTRODUCTION

In the past biological studies, developments in epigenetics have significantly changed our understanding of the mechanisms that regulate physiological or pathological signals (1,2). Aberrant epigenetic changes are important factors in the occurrence and development of diseases, which can influence multiple biological processes (3,4). Chromatin regulators (CRs) are enzymes with specific domains. They are indispensable upstream regulators in epigenetics regulating epigenetic patterns, and thus, managing many target genes (5). CRs are generally grouped into three major classes in accordance with their regulatory roles in epigenetics: histone modifiers, DNA methylators, and chromatin remodelers (6–8). Chromatin remodeler is a specific-type CR that causes aberrant epigenetic modifications by disrupting the connection between DNA and nucleosomes, disturbing and replacing nucleosomes, or removing nucleosomes from chromatin (6,7,9). The histone modifiers and DNA methylators have the functions of encoding and decoding sundry modifications on the cytosine and histone residues and are generally divided into erasers, writers, and readers (6,7,9). For instance, numerous studies showed that the presence of many dysregulation CRs (e.g., UHRF1, DNMT1, EZH2, BMI1 and HELLS) might lead to an abnormal epigenetic landscape in retinoblastoma (RB), which might be associated with tumorigenesis and malignant progression (10–13). Importantly, CRs indirectly affect the expression of downstream target genes by regulating the modifications of histones or DNA in regions of regulatory elements associated with gene transcription. For example, EZH2 mediates transcriptional repression by regulating the dimethylation and trimethylation of H3K27 on the promoters/enhancers of downstream target genes and is involved in stem cell differentiation, development, and maintenance of self-renewal (14,15). In addition, the disruption of upstream signaling further affects CR activity and changes the expression of downstream target genes. For instance, the increased expression of FOXO1 can activate RB1, a chromatin regulator, which causes senescence in hepatocellular carcinoma cells (16,17). These studies indicated that CRs could change the histone/DNA modifications in specific regions of chromatin or change chromatin structure, affecting the expression of downstream genes. Meanwhile, CRs are also regulated by the upstream signals, and their activity can be further affected. Moreover, they play an indispensable role in the occurrence and development of diseases as well as various biological processes of cells.

Several databases associated with CRs have been developed by previous studies, which provide worthwhile information on the biological functions of CRs. For example, CR2Cancer (18) comprises annotated data and abnormal patterns of CRs in human cancers to explore the function of CRs based on literature mining and high-throughput data analysis. FACER (4) provides three layers of information on functional CRs related to cancer: basic information (e.g., biological function and protein complexes), epigenetic categories, and cancer types, and information on seven abnormal features. The chromatin immunoprecipitation sequencing (ChIP-seq) technique has become a vital approach to identify the functions of CRs and their target genes. Especially, CR-Cistrome (9) stores 49 CRs and 1,566 ChIP-seq datasets associated with CRs, which can be used to analyze genome cis-acting elements affected by CRs. Nevertheless, a database comprising a large number of CRs and providing detailed regulatory information on CRs has not been established. Recently, the rapid accumulation of CR-related ChIP-seq data provided an opportunity to discover CR regulatory mechanisms and identify downstream target genes of CRs, thus constructing a complete CR-related regulatory network. However, substantial time and computational resources were required to process and analyze the ChIP-seq data for biologists. Consequently, a comprehensive resource of CRs needs to be established urgently for collecting, integrating and processing these data, which can provide rich annotated information on CR upstream and downstream regulatory analyses as well as CR-related analysis functions.

**Figure 1.**
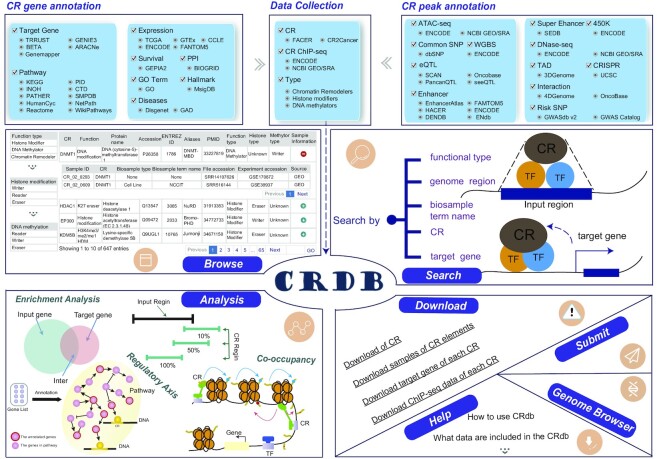
Database content and construction. CRdb collected and processed a great deal of CR-associated ChIP-seq data and provided abundant multi-omics annotations for CRs. Meanwhile, CRdb contained numerous functions and analytical tools for browsing, searching, downloading and visualizing CRs.

Keeping this in mind, the present study established the CRdb database (http://cr.liclab.net/crdb/), which aimed to record massive available resources for anthropic CRs and provide substantial annotations and analysis functions of CRs. The current version of the CRdb database comprised a total of 647 CRs and more than 2,500 CR-associated ChIP-seq samples from more than 300 human tissues and cell types, while the ChIP-seq samples are nearly twice as many as the other databases. This study analyzed the ChIP-seq data and identified CR-binding regions. CRdb emphatically provided the circumstantial and numerous (epi) genetic annotations in CR-binding regions by processing and integrating large-scale high/low-throughput experimental data, including super-enhancers, enhancers, whole-genome shotgun bisulfite sequencing (WGBS), single-nucleotide polymorphism (common SNP), risk SNPs, linkage disequilibrium (LD) SNPs, expression quantitative trait locus (eQTL), topologically associated domain (TAD), chromatin accessibility (ATAC), DNase I hypersensitive signal (DHS), clustered regularly interspaced short palindromic repeats (CRISPR) and 3D chromatin interactions. The downstream target genes of CRs could also be found by five calculative methods in CRdb. Furthermore, CRdb supported upstream regulatory information and various functional annotations of CRs, including Gene Ontology (GO) (19) terms, pathways, protein-protein interactions (PPIs), cancer hallmarks, survival, disease information and gene expression. In particular, the database embedded four functions of CR regulatory analyses for users exploring the potential biological functions of CRs. CRdb is a convenient and friendly resource for users to browse, inquire, analyze and visualize the basic and exegetical information (Figure 1). CRdb can become a serviceable and powerful platform to explore the underlying functions and regulatory mechanisms of CRs in diseases and biological processes.

## MATERIALS AND METHODS

### CR acquisition and classification

We collected a list of human CRs from the database and literature (4,8,18,20–22) and searched the corresponding high-quality research literature as references of CRs (Supplementary Material). Importantly, based on the biological function of CRs, we divided all the CRs into three categories: histone modifiers, DNA methylators, and chromatin remodelers. Notably, only the CRs with certain epigenetic functions were reserved. As a result, a total of 647 CRs were obtained and divided into three categories (463 histone modifiers, 26 DNA methylators, 116 chromatin remodelers and 42 two-type regulators) based on their functions in epigenetics. Specifically, histone modifiers and DNA methylators were correspondingly divided into three regulatory types: readers, writers, and erasers. Further, all human CRs were annotated with their full name, aliases and chromosome location.

### CR ChIP-seq data collection and identification of binding regions

CR ChIP-seq samples were identified from NCBI Gene Expression Omnibus (GEO) (23) and Encyclopedia of DNA Elements (ENCODE) (24). For ENCODE, we used the following filters to obtain the metadata information: Assay ‘ChIP-seq’; Organism ‘Homo sapiens’; and Available file types ‘fastq’. Then, manually curated and reserved CR ChIP-seq samples. In GEO, the query ‘ChIP-seq’, ‘Homo sapiens’ and not ‘ENCODE’ were used to obtain identifiers (GSM IDs) from the SRA database. In total, we processed 2,591 CR-associated ChIP-seq datasets from GEO and ENCODE. To identify the CR binding regions, we first used Bowtie2 (v2.4.2) (25), one of the most commonly alignment methods on the market today, to map the ChIP-seq reads to the human reference sequence (hg38) which downloaded from the UCSC genome bioinformatics (26). Then, we ran SAMtools (27)-MACS2 (v2.2.7) (28) for each Bowtie2-generated sequence alignment file (in .sam format) to peak calling.

The quality control for the CR ChIP-seq data was important because CR ChIP-seq samples were obtained from different source. For each ChIP-seq sample, we provided seven different quality metrics, such as number of sample reads within analyzed chromosomes, the estimated fragment length by cross-coverage method, SSD score RIP, and so on using the ChIPQC. We provided a link to display the detailed ChIP-seq quality in each CR ChIP-seq sample page for the convenience of the users. This quality control was used to evaluate the data for users, not strictly for classifying samples as pass or fail.

### Annotations of CR regulatory element

CRs are crucial regulators of gene transcription, which can link regulatory cues from enhancers to promoters. Numerous genomic annotations on the regulatory element regions were collected as regulatory cues of CRs, including enhancers, super-enhancers, accessible chromatin, 3D chromatin interaction, DNA methylation, CRISPR and TAD. Besides, SNP on the CR-binding regions might affect the binding of various regulators, affecting the activity of the regulatory elements. Therefore, the information on common SNP, risk SNP, LD SNPs and eQTL was collected.

#### Enhancers

For obtaining the regions of enhancers, we collected enhancer datasets from ENdb (29), EnhancerAtlas (30), FANTOM5 (31), ENCODE (24), HACER (32) and DENdb (33). Of which, enhancers collected from some databases were identified by high-throughput experimental sequencing, such as EnhancerAtlas (24) and FANTOM5 (31). In addition, experimentally verified enhancers were collected from ENdb (29), which was the previous work of our research group. Overall, CRdb comprised 120,626,280 enhancers.

#### Super-enhancers

The super-enhancer datasets were obtained from SEdb (34), which was published by our laboratory. We came to its super-enhancer region by analyzing the distribution of the H3K27ac modification signal in the ChIP-seq data of CR. Bowtie2 (v2.4.4) (25) and MACS2 (v2.2.7) (28) were performed to identify enhancer constituents. The ROSE (35) algorithm was then used to identify the super-enhancer region. Finally, CRdb contained 1,167,518 human super-enhancers.

#### Accessible chromatin

Emerging evidence has revealed that CR-binding regions have high chromatin openness scores, which are useful for recruiting TFs and other transcription regulators. ATAC-seq and DNase-seq are extensively used to identify accessible chromatin regions across the genome (36–38). We downloaded DNase-seq datasets from the public database ENCODE (24) and ATAC-seq datasets from NCBI GEO/SRA (23). We used the workflow of Bowtie2 (25)-SAMtools (27)-MACS2 (28) for each dataset to identify the accessible-chromatin regions (Figure 1 and Supplementary Table S1). Besides, the pipelines from ATAC-seq datasets were the same as pipelines of DNase-seq data processing (Figure 1 and Supplementary Table S1). Finally, CRdb involved 199,881,570 accessible chromatin regions.

#### TAD/3D chromatin interaction

A complex relationship existed between the CR-binding regions and TAD/3D chromatin interaction. The disruption of chromatin interactive structure could lead to the dysregulation of downstream genes. To better understand whether CR-binding regions were TADs, we obtained TADs for 21 tissue types from 3D Genome Browser (39), and the annotated information of 72,019 sets of TAD and related details of CR binding chromatin regions (Figure 1 and Supplementary Table S1). Additionally, we gained 34,342,925 sets of 3D chromatin interaction data (e.g. Hi-C, ChIA-PET 3C, 4C and 5C) from the 4DGenome (40) and OncoBase (41) (Figure 1 and Supplementary Table S1).

#### Common SNP/LD SNPs/risk SNP/eQTL

A total of 37,302,778 human common SNPs were acquired from dbSNP (42). VCFTools (V0.1.13) (43) was used to filter out SNPs with minimal allele frequency (MAF) <0.05 (Figure 1 and Supplementary Table S1). LD was calculated using phasing genotypic information accompanying the 1000 Genomes Project phase 3. Here, we used plink (v1.9) (44) to identify LD SNPs in five races (East Asia, South Asia, Europe, Admixed America and Africa). Further, 351,728 human risk SNPs were downloaded from GWAS Catalog (45) and GWASdb (v2) (46); meanwhile, we collected 2,886,133 human eQTL from SCAN (47), seeQTL (48), PancanQTL (49) and Oncobase (41) (Figure 1 and Supplementary Table S1).

#### CRISPR

DNA sequence that targets the CRISPR RNA sequences and the transcriptional region within 200 bp of the genomic region was shown by annotation of the CRISPR/Cas9 target sites (26,50). The CRISPR/Cas 9 target site was downloaded from the UCSC (26) and then predicted using the CRISPOR tool (51). It was next mapped to each CR regulatory region, which benefited to the design, evaluation and cloning of CRISPR/Cas 9 system-directed sequences on the CR regulatory region.

#### DNA methylation

Overall, 32,099,123 methylation sites were downloaded from 450K array of ENCODE (24). We also obtained 176,535,821 WGBS datasets from ENCODE (24) (Figure 1 and Supplementary Table S1).

### Identification of CR-associated genes

CRs maintain chromatin structure and function by binding or catalyzing histone modifications, thereby affecting the transcriptional process of their downstream target genes. For identifying the downstream target genes of CRs, five different methods were used: (i) the BETA algorithm (52) was directly used to predict the downstream target genes of CR by integrating ChIP-seq data. (ii) Genemapper (35), a python script of ROSE, was used to annotate the downstream target genes of the CR ChIP-seq regions. ROSE (35) has three strategies to calculate the target genes: closest, proximal, and overlap. (iii-iv) GENIE3 (53) and ARACNe (54) were used to identify human CR target genes by inputting the cancer expression matrix from The Cancer Genome Atlas (TCGA) (55). (v) We extracted the experimentally validated CR-downstream target gene pairs from TRRUST (v2) (56) (Figure 1 and Supplementary Material).

### Functional annotations of CRs

Except for the annotations of CR regulatory elements, the additional annotations of CRs (e.g., GO, Hallmarks, the relevant pathways of CRs, expression, disease, and survival) were obtained from various sources for the purpose of elucidating the biological function of CRs. The experimentally-supported CR-disease correlations in human were obtained from GAD (57) and DisGeNET (58) (Figure 1). Simultaneously, we extracted the PPIs of CRs from BIOGRID (59). We combined the details of 2,169 human pathways from a 10-pathway database containing CTD, EuRBPDB, HumanCyc, INOH, NetPath, PANTHER, PID, Reactome, SMPDB, WikiPathways and KEGG (60–71) (Figure 1). Furthermore, we acquired 10 cancer hallmarks from Hanahan and Weinberg (72) and 31 GO terms from Gene Ontology (19) (Figure 1). We performed the survival analysis on CR and obtained the TCGA cancer survival map using python package of GEPIA2 (73) (Figure 1).

## USER INTERFACE

**Figure 2.**
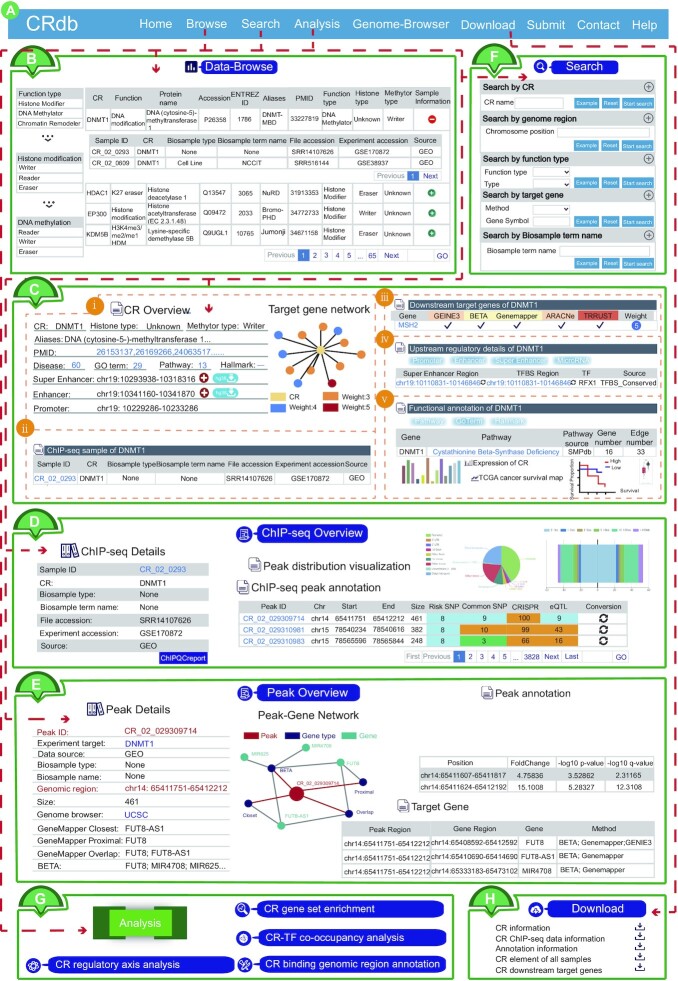
Main functions and usage of CRdb. (**A**) The navigation bar. (**B**) Data browse for CRs. (**C**) Search results containing CR overview, ChIP-seq information, downstream target genes, upstream regulatory details, annotation, expression and TCGA cancer survival map. (i) CR overview and the network chart consisting of CR and its regulated genes. The regulated genes labeled by weightness of prediction. (ii) A detailed interactive table about ChIP-seq information, including Sample ID, CR name, Biosample type, Biosample term name, File accession, Experiment accession and Source. (iii) A tabulation about CR-downstream target genes obtained by five different methods (BETA, Genemapper, ARACNe, GENIE3 and TRRUST). (iv) The table describes the information on the upstream regulatory regions of CR (containing enhancer region, super-enhancer region and promoter region) and information on microRNA with a targeted relationship to the CR. (v) The functional annotation of CR, Proteins that interact with CR, TCGA cancer survival map of CR and expression of CR. (**D**) Interactive table with detailed information about ChIP-seq samples of interest and the visualization of peak annotation. (**E**) The detailed interactive table of annotation information and ChIP-seq peak associated genes that are identified through five strategies. Network diagram about these regions is displayed. (**F**) Five inquiry modes are available. (**G**) Online analysis tools. (**H**) Data download.

CRdb is a powerful and comprehensive platform for users to browse, query, analyze, visualize and download the basic and annotated information about CR of interest, and for biologists to explore the underlying functions and regulatory mechanisms of CR in disease and biological processes (Figures 1 and 2A).

### User-friendly browsing

The ‘Data-browse’ page in the CRdb database allows users to quickly search for CR using two different methods. Users can retrieve the corresponding CR by selecting ‘Function Type of CR’ (including histone modifier, DNA methylator, and chromatin remodeler), ‘Histone modification’ and/or ‘DNA methylation’ (including reader, writer, eraser and unknown) via the navigation bar on the left side of the page. Moreover, the right side of this page is an interactive table presenting the retrieval results from the alphabetical sorting function, and each CR in the table is hyperlinked to an individual CR page for further information (Figure 2B). Additionally, users can obtain the ChIP-seq sample information for each CR by clicking the ‘the plus sign’ in the sample information column of this table (e.g., Sample ID, CR, Biosample type, Biosample term name, File accession, Experiment accession and Source). By selecting a CR name in the search results table, users can obtain detailed information about the CR, including five modules as follows:

- **CR overview**. Users can obtain general information on the searched CR, including the CR name, uniport accession, protein names, the aliases, functional type of CR (histone modifiers, DNA methylators or chromatin remodelers), number of diseases, number of pathways, upstream super-enhancer/enhancer/promoter region of CR, PMID of the study related to CR, and network consisting of CR and its regulated genes (Figure 2C_i_).
- **ChIP-seq samples of CR**. We used a list to present the basic information about these CR ChIP-seq smaples, including Sample ID, CR name, Biosample type, File accession and Source (Figure 2C_ii_). Users can select a ‘Sample ID’ of interest to view the details about the ChIP-seq sample (including ChIP-seq overview, ChIP-seq peak annotation and visualization). For ChIP-seq peak annotation, the interactive table represents the peak ID, genome location, size, and detailed genetic and epigenetic information in the CR ChIP-seq regions. CRdb visualizes ChIP-seq peaks through ChIPseeker (74), providing the distribution of CR-binding peaks, including TSS, 5'UTR, 3'UTR, introns or exons and so on (Figure 2D). In addition, users can obtain elaborate information on the CR ChIP-seq area by selecting the ‘Peak ID’. On the peak details page, CRdb exhibits the peak overview, annotated information (enhancers, super-enhancers, risk SNPs, common SNPs, LD SNPs, eQTLs, DHSs, ATAC, 3D chromatin interactions, WGBS, TAD and CRISPR) and the peak-gene network calculated using BETA and GeneMapper, where it is worth mentioning that we also provide regional information for its target genes (Figure 2E).
- **Downstream target genes of CR**. In detail, CRdb displayed a list of CR-downstream target genes predicted by five diverse methods (BETA, Genemapper, ARACNe, GENIE3 and TRRUST). In particular, the target gene weightiness of CRs, as a measure of the potential relationship between the CR and the target genes, was defined by calculating the sum of the prediction. This section could help researchers in judging the regulatory relation between CR and the target genes from multiple perspectives (Figure 2C_iii_).
- **Upstream regulatory details of CR**. For the details of upstream regulation, the table describes the information on the upstream regulatory regions of CR (including promoter region, enhancer region and super-enhancer region) and information on microRNA with a targeted relationship to the CR. By integrating the upstream and downstream regulatory information, users can establish a regulatory axis of upstream regulatory element-CR-downstream target genes (Figure 2C_iv_).
- **Additional information on CRs**. The extra information included CR-related PPI, functional annotation, TCGA cancer survival map, disease information and expression (see Materials and Methods) (Figure 2C_v_).

### Genome browser

CRdb provides a personalized and powerful genome browser through JBrowse v2.0.0 that helps users visualize regulatory information about human CRs by customizing traces of interest, including CR ChIP-seq regions, genes, SNP, super-enhancers, TAD, and so on.

### Search interface for CRs

On the Search page, CRdb offers five query methods: ‘Search by CR’, ‘Search by CR function type’, ‘Search by target gene’, ‘Search by genomic region’ and ‘Search by Biosample term name’ (Figure 2F).

In the *‘Search by CR’* query, users can query multiple CRs via different IDs, including symbol, ENTREZ GENE ID, protein name and uniprot accession, and so on. Users can get a result table for the CRs of interest, showing the basic information of CRs. By selecting a CR name in the result table, users can obtain detailed information about the CR. In the ‘*Search by genome region*’ query, by inputting a genomic region, CRdb identifies the CR ChIP-seq peak regions overlapping with the submitted region, and then outputs a result table. Specifically, even if the ChIP-seq peak region of the CR intersects with only one base of the user-submitted region, that will also be displayed in the result table. Users can select ‘Peak ID’ in the result page, obtaining detailed information about the peak. In the ‘*Search by target gene*’ query, by submitting a gene of interest and selecting a target gene prediction method (BETA, Genemapper, ARACNe, GENIE3, TRRUST or all), and CRdb will extract all the CRs that may regulate this gene in the upstream region in the outcome table. Users can also select ‘the function type of CR’ and ‘the idiographic type’ in the ‘*Search by CR function type*’ query. CRs are divided into three major classes (histone modifiers, DNA methylators and chromatin remodelers) based on the regulatory roles in epigenetics, and then histone modifiers and DNA methylators are further grouped into erasers, writers and readers according to their modification (9,75,76). CRdb displays the specific information about such CRs on the result page, which contains CR name, aliases, ID, function, type and PMID. Users can select the CR to further view the information of CR. Users can input the biosample term name in the ‘*Search by Biosample term name*’ query. On the result page, the user can further view the ChIP-seq sample information associated with CRs, including sample ID, CR, Biosample type, Biosample term name, File accession, Experiment accession, and Source. By clicking on the CR-related ChIP-seq sample ID, users can obtain the details of the ChIP-seq sample (e.g. ChIP-seq overview, ChIP-seq peak annotation and peak distribution visualization).

### Online analysis tools

We designed and provided four analytical methods for biologists to elucidate the functional mechanisms of CR (Figure 2G). Gene set enrichment analysis is one of the most common and effective methods that can be used to identify whether a set of genes of interest is significantly related to biological processes and functions (Supplementary Material). Therefore, we designed ‘CR gene set enrichment analysis’ function, which could determine whether a set of genes of interest are regulated by CRs. In the *‘CR gene set enrichment’* analysis, users can submit a set of genes and set the threshold value of P-value/FDR, and then CRdb identifies the significantly enriched CR using the hypergeometric test. The background set for enrichment analysis has two options (including experimental validation and high confidence) that enable users to select different background sets for enrichment analysis. The experimentally validated CR target gene set obtained from TRRUST was used as the default background set for enrichment analysis, so as to improve the reliability of enrichment analysis. In the ‘*CR-binding genomic region annotation*’ analysis, when the user inputs the genomic region of interest (in .bed format), the CRdb identifies the CR that can be combined with the input genomic region. Particularly, users can customize the minimum proportion of the input genome region overlapping with the CR region in the analysis. After choosing one of CR, users can acquire the comprehensive annotation for its overlapping peaks. In the ‘*CR-TF co-occupancy analysis*’, users can obtain the number of co-binding peaks for the CR-TF pairs by inputting multiple CRs and TFs. Similarly, users can also input CR(s)/TF(s) to find all TFs/CRs that have a co-localization relationship with it. By clicking on the ‘Details’ button, users can obtain the detailed result containing Peak ID, Sample ID, Sample name, region, *P*-value, score and the sequence. In the ‘*CR regulatory axis analysis*’, users can submit a gene set of interest and select at least one pathway database (e.g., KEGG), while also selecting the *P*-value/FDR, and CRdb will identify pathways significantly enriched for these genes using hypergeometric tests. Moreover, it can identify CRs on the downstream of pathway, thus providing regulatory information about these CRs.

### Data download

All CR-related files (including CR information, CR ChIP-seq data information and CR-downstream target genes) are supported for download on the ‘Download’ page. Users can promptly retrieve and download associated information (Figure 2H). Meanwhile, CRdb also supports the packaged download of CR ChIP-seq samples and downstream target genes of CRs. Notably, CRdb provides their packaged download classified based on different functional types of CRs (histone modifier, chromatin remodeler and DNA methylator). In addition, CRdb provides the global CR regulatory network downloads and the download of annotation information (e.g., PPI of CR, disease, pathway, enhancer, super-enhancer, eQTL, common SNP, risk SNP, DHS, TAD and so on). Besides, users can click on the ‘Export’ button to export all the results of interest in the CR detail pages, search result pages and analysis result pages.

### Data submission

Users can share their CR-related ChIP-seq data in the CRdb. For ensuring the data quality control, CRdb recommends that users submit the corresponding GEO/SRA series number or the corresponding accessible URL for the raw data on the ‘Submit’ page. Moreover, the submitter is required to fill in the relevant information (e.g., Biosample type, Biosample term name, File accession, Experiment accession, Storage location and Submitter information) for the submitted data, as well as the user's e-mail, to facilitate subsequent contact. We will determine the data-update time dynamically based on the sample size submitted by the user, ensuring the timely release of the data.

### Case study

#### Case exploration of EZH2

**Figure 3.**
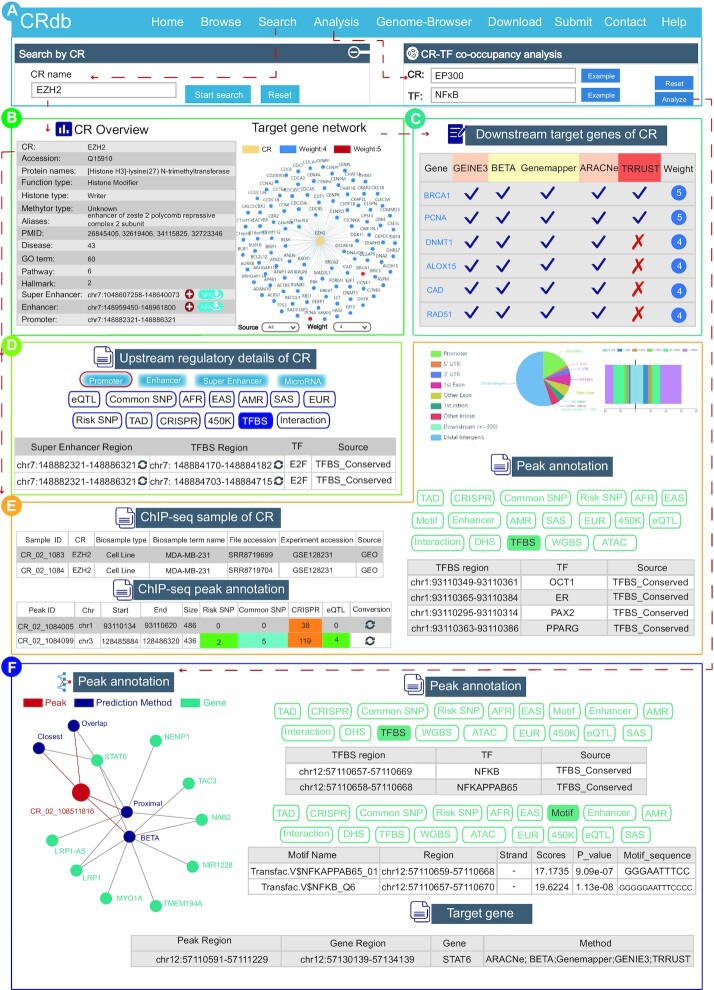
Validation results about case exploration. (**A**) The navigation bar of CRdb and the interface for inputting case study. (**B**) EZH2 overview and EZH2 target network. (**C**) Downstream target genes of EZH2. (**D**) Upstream regulatory information of EZH2. (**E**) ChIP-seq information of EZH2, detailed information and visualization of peak annotation about CR_02_1084005. (**F**) Peak-target gene network, detailed interactive table of annotation information (TFBS) of CR_02_108511816 and specific information about downstream target genes of EP300.

EZH2 has been demonstrated to be a member of the Polycomb-group (PcG) family, forming multimeric protein complexes, which are involved in maintaining the transcriptionally repressive state of genes over successive cell generations. The EZH2 expression has been found to be altered in multiple cancers, such as breast cancer, esophageal cancer, liver cancer and so on. The expression level of EZH2 is closely related to the malignancy degree, treatment and prognosis of the tumor (77). EZH2 was used as an idiographic example in this study to explain how to use CRdb and attest to its practicability (Figure 3A). In the result page returned by CRdb, we first obtained the overview of EZH2 information, containing basic information (uniport accession, function type, aliases, literature research PMID, pathway and disease), target gene network, and information on the upstream regulatory element (promoter, enhancer, and super-enhancer) (Figure 3B). The detailed weightiness of the target genes was shown in the table of ‘Downstream target genes of EZH2’. Notably, some acknowledged EZH2 target genes (such as BRCA1, RAD51, and PCNA) were identified as extremely persuasive targets with high weight, indicating that the CRdb had the potentiality and confidence in identifying the target genes of CR (78–80) (Figure 3C). Second, the upstream regulatory information showed that the CRdb provided a wealth of genetic annotation information, including eQTL, common SNP, risk SNP, CRISPR, TAD and 450K. Here, we found that EZH2 was regulated by the transcription factor E2F binding to its upstream region, which was consistent with the result in the study of Bracken AP (81) (Figure 3D).

Furthermore, the CRdb database was quite competent at revealing downstream regulatory cues for EZH2 based on the ChIP-seq data. The information on the ChIP-seq samples of EZH2 could be acquired from the result page, including Sample ID, CR name, Biosample type, Biosample term name, File accession, Experiment accession and Source. Among these, both the sample file accession and the experimental accession could be downloaded for free through the hyperlink (Figure 3E). Further, the detailed annotation of the ChIP-seq dataset was obtained by clicking on the sample ID (e.g. CR_02_1084, a sample of breast cancer cell line), and then a detailed page containing the following information was acquired: (i) a brief overview of the EZH2 ChIP-seq dataset; (ii) ChIP-seq peak annotation information, containing SNP, CRISPR, eQTL and others and (iii) genomic distribution of EZH2 ChIP-seq peaks, including the following nine regions: promoter, 5′UTR, 3′UTR, first exon, exon, first intron, intron, downstream and distal intergenic regions (Figure 3E). Here, surpassing 50% of the EZH2 binding peaks were significantly enriched in the distal intergenic region (Figure 3E). In addition, CRdb also exhibited details of each peak of EZH2. The Peak ID (CR_02_1084005) was selected to obtain the peak overview, the peak-gene network calculated using BETA and Genemapper, the annotation information and the region of their target gene. We found that estrogen receptor (ER) was occupied at this EZH2 peak in the module ‘TFBS’ (Figure 3E). Indeed, Shi et al demonstrated that the physical interaction between EZH2 and ER stimulated the gene transactivation of estrogen and Wnt pathways, hence phenotypically promoting the breast cancer cell cycle progression (82). Similarly, we also found that octamer transcription factor 1 (OCT1) was taken up at the same peak in the module ‘TFBS’. Interestingly, Hwang-Verslues *et al.* revealed that TF OCT1 occupied the binding site of EZH2 in an anoxic environment, a common condition of tumors, activating the expression of epithelial-mesenchymal transition (EMT) genes (83). This declared that the synergistic effect or the mutually exclusive effects could be found in TFBS modules of peak annotation. In addition, we also observed that PAX2 is occupied the same peak. The cooperation between PAX2 and EZH2 has been confirmed to be abnormally expressed in ovarian cancer, whereas not reported in breast cancer (84) (Figure 3E). Accordingly, we speculated that EZH2 might cooperate with PAX2 in breast cancer, which needs to be further investigated. These results confirmed that CRdb could be used to explore the underlying regulatory mechanisms of CRs in disease or biological processes.

#### Case exploration of online analysis

Atherosclerosis is a chronic inflammatory disease characterized by lipid deposition in the arterial wall. Atherosclerotic cardiovascular disease has been a major cause of morbidity and mortality worldwide (85–88). Increasing evidence shows that the epigenetic processes play a crucial role in the occurrence and development of atherosclerosis (89,90). EP300 has been demonstrated to be a KAT3 family member, not only as a histone acetyltransferase but also as a key co-factor for many cardiovascular diseases (91,92). Transcription factor nuclear factor kappa-beta (NF-ĸB), is a driver of inflammation and plays an essential role in the pathogenesis of atherosclerosis by regulating many pro-inflammatory genes related to atherosclerosis (93–95).

In the ‘CR-TF co-localization analysis’, EP300 and NFKB1 were used to explore the synergistic regulatory mechanisms and further verify the function of the CRdb (Figure 3A). We observed from the result page that 6502 regions were shared in the genome (Figure 3F). By clicking the ‘Detail’ button, a result page about co-localization region information could be acquired, which included the Peak ID, Sample ID, TF, region information, scores, *P*-value, sequence information, type and cell line. Particularly, we found that EP300 cooperated with NF-ĸB was similar in the two HUAEC (Human Umbilical Artery Endothelial Cells) samples that are most associated with atherosclerosis (Figure 3F). We selected the peak ID (CR_02_108511816), the most significant peak of NF-ĸB binding, to further view the peak details of the EP300. NF-ĸB was found to occupy the EP300 peak in the TFBS module. Moreover, STAT6 was also identified as a target gene of the EP300 peak (CR_02_108511816) (Figure 3F). STAT6 is a member of the family of activators of signal transducers and activators of transcription (STATs). It activates gene transcription in response to extracellular cytokine signals and growth factors to induce epigenetic alterations, thus regulating the expression of genes involving the inflammatory response (96,97). In addition, reduced EP300 has been shown as a key mechanism that can inhibit the transcription of STAT6 (98). Meanwhile, EP300 is known to acetylate NF-ĸB by binding to it, causing an increase in the combination between DNA and NF-ĸB, thus promoting transcriptional activation (88). Consequently, we concluded that the synergistic effect of EP300 and NF-ĸB could regulate the expression of STAT6, thereby affecting the occurrence, development and deterioration of inflammation. These results further demonstrated that CRdb provided feasibility and practicality for the researchers’ finer experimental design in CR studies.

## DISCUSSION

CRs are a class of specialized domain-containing enzymes that recognize, form and maintain an epigenetic state in a cell-context-dependent manner. It has been proved that the abnormal expression of CRs is related to multiple biological processes, and the dysregulation of CRs can lead to the occurrence, progression or deterioration of various diseases, such as cancer, inflammation and so on. Meanwhile, CR transcriptional activity was also governed by numerous of upstream regulators, such as TFs and cis-regulatory elements. Large number of studies demonstrated that CRs can regulate panoramic epigenetic patterns to influence multiple target genes simultaneously. Aberrant CRs provided disease cells an efficient mechanism to rewire pathological transcriptional regulatory circuits. Thus, deciphering CR-mediated transcriptional regulatory network is essential for understanding the molecular mechanisms of pathological and physiological processes. For instance, EP300 is largely acknowledged in cancer therapy for its advanced roles in SE organization and oncogenes transcription regulation. CR-Cistrome (9), CR2Cancer (18) and FACER (4) have sorted out basic information, annotated information, as well as the predicted targets of plenty of CRs. However, an integrative and detailed regulatory database of CRs has not yet been established, further affecting the progress of the study of CRs. Simultaneously, the rapid accumulation of CR-related ChIP-seq data allowed us to find downstream target genes of CRs and the regulatory information on CRs upstream and build a complete CR-related regulatory network. Consequently, we developed CRdb, a comprehensive and powerful database, recording the extensive available CRs resources and providing abundant annotation and analysis. The current version of CRdb integrated and processed a total of 647 CRs and 2,591 CR-related ChIP-seq data from more than 300 human tissues and cell line types. Contrary to the other resources, CRdb displayed multiple advantages, such as CR ChIP-seq data scale and abundant (epi) genetic annotations in CR-binding regions (Table 1 and Supplementary Tables S1 and S2). The CRdb database also supported numerous functional annotations and upstream regulatory information for CRs. And the CR-downstream target genes were identified by several approaches, based on ChIP-seq, expression and experimental validation. In conclusion, CRdb proved to be a valuable resource for researching CRs regulatory cues.

**Table 1.** Comparison of CR associated information in CRdb with other databases

Attribution	CRdb	CR Cistrome	FACER	CR2Cancer
Functional CR number	647	49	519	429
CR ChIP-seq sample number	2,591	1,566	−	1,359
Quality control	Reads, Dup%, FragLen, RelativeCC, SSD, RIP%	Reads	−	−
Peak annotation visualization	√	−	−	−
Upstream annotation	Promoter, Enhancer, Super-enhancer, MiRNA	MiRNA	MiRNA	MiRNA
Region annotation	Enhancer, Super-enhancer, Accessible chromatin, Chromatin Interaction, TAD, Common SNP, LD SNP, risk SNP, eQTL, CRISPR/Cas9 target site, Methylation site	−	Common SNP, LD SNP, risk SNP, Methylation site	Methylation site
Functional annotation	GO Term, Cancer Hallmark, Survival, PPI	−	Cancer Hallmark, Survival, PPI	GO Term, Survival,PPI
Pathway sources	KEGG, Reactome, PANTHER, SMPDB, NetPath, PID, HumanCyc, CTD, WikiPathways, INOHstarBasev2.0, EuRBPDB	−	−	KEGG, Reactome
Expression sources	TCGA, CCLE, GTEx, ENCODE	−	TCGA	TCGA, CCLE, HPA
Disease information sources	DisGeNET, GAD	√	TCGA	√
Strategies of CR associated genes	BETA, Genemapper (Closest, Overlap and Proximal), GENIE3, ARACNe, TRRUST	−	−	ARACNe, BETA
Online analysis	√	−	−	−

The CRdb is the first comprehensive resource focusing on systematically revealing the regulatory mechanisms of CRs (Table 1 and Supplementary Table S2). Researchers can probe CR regulatory information and accomplish CR-associated regulatory analyses. The advantages of the CRdb are reflected in many aspects, including: (i) integrative annotations about genetics and epigenetics in ChIP-seq regions related to CRs; (ii) identification of downstream target genes of CRs by five methods; (iii) comprehensive annotation information related to CR upstream regulatory regions; (iv) valuable online analytical tools; (v) five search methods to access CRs; (vi) user-friendly browsing and (vii) full-featured detail pages including CR different functional annotations, disease information and survival analysis tool.

In the current version of CRdb, we integrated and processed a great number of CR-related ChIP-seq datasets and embedded abundant annotation information on CRs, which filled the gap of the precise identification of CR-related regulatory mechanisms in complex diseases or biological processes. CRdb showed numerous advantages of information and functions compared with other CR-associated databases (Table 1 and Supplementary Table S2). Nevertheless, we found that some CR regulatory information was not supported due to the deficiency of ChIP-seq data. We will follow up on any updated ChIP-seq data and its annotation information and we will add the updated results to the database regularly in the future updates. For exploring the functions and regulatory mechanisms of CRs, we will continue to add more precise methods for inferring regulatory networks mediated by CR. In conclusion, CRdb is a powerful and comprehensive platform for users to browse, query, analyze, visualize and download basic and annotated information on CRs. We believe CRdb will be an advantageous and useful platform for probing the underlying functions and regulatory mechanisms of CRs in disease and biological processes.

## DATA AVAILABILITY

The research community can access information freely in the CRdb without registration or logging in. The URL for CRdb is http://cr.liclab.net/crdb/.

## Supplementary Material

gkac960_Supplemental_Files
