# Supplementary material for: CRdb: a comprehensive resource for deciphering chromatin regulators in human
Source: Nucleic Acids Res. 2022 Nov 1;51(D1):D88–D100. doi: 10.1093/nar/gkac960 (PMC9825595; doi:10.1093/nar/gkac960)
Supplement: gkac960_Supplemental_Files [file gkac960_supplemental_files.zip › Supplementary Materials.doc]

**CRdb: a comprehensive resource for deciphering chromatin regulators in human**

**Supplementary material**

**Supplementary method**

**CR acquisition and classification**

To ensure the timeliness and comprehensive coverage of CRs in our database, we manually collected the list of CRs from the database and a large number of literatures ([1-6](#_ENREF_1)). In this study, nearly 1,700 articles were manually curated and independently and repeatedly verified by six biologists, thus we obtained CRs with certain epigenetic functions. In addition, we searched corresponding high-quality literatures related to each CR as references for further study. Based on the biological functions, CRs are classified into three broad categories: histone modifiers, DNA methylators, and chromatin remodelers. Specifically, histone modifiers and DNA methylators can be corresponding divided into three types of regulation: readers, writers, and erasers. In summary, CRdb contained a total of 647 chromatin regulators (463 histone modifiers, 26 DNA methylators, 116 chromatin remodelers and 42 two-type regulators).

**Identification of CR-associated genes**

We used five different methods to identify CR downstream target genes.

(I) We used the BETA algorithm([7](#_ENREF_7)) to identify the downstream target genes of CRs. BETA is a software package based on the distance of binding sites and transcription start sites, and it can directly infer downstream target genes by integrating CR-associated ChIP-seq data. Genes within 100 kb of CR-binding region were considered as downstream target genes by BETA. BETA minus([7](#_ENREF_7)) was used in this study to identify downstream target genes by inputting human CR ChIP-seq datasets (in “.bed” format).

(II) Genemapper([8](#_ENREF_8)), a python script of ROSE, was used to identify the downstream target genes of CRs from human CR-related ChIP-seq datasets. ROSE can identify downstream target genes by calculating the proximity of CR-binding regions to genes, yielding the proximal genes, overlap genes and closet genes. “Proximal genes” refer to genes within 50 kb from the CR-binding region, “Overlap genes” refer to genes overlapping with the CR binding site region on the chromosome, and “Closet genes” refer to genes closest to the CR-binding region. The relationship between CRs and genes, which has already been mentioned, was retained in this study.

(III) In systems biology research, reverse-engineering usually uses transcriptomic data, proteome data and other quantitative data in biology to infer the corresponding regulatory network or regulatory patterns, and reveal the corresponding regulatory rules. GEINE3([9](#_ENREF_9)) is a reverse-engineering-based approach that extracts gene regulatory networks from a large amount of expression data. This process decomposes the problem of inferring a network of size “n” into *n* different feature selection problems, with the goal of determining the regulators of a gene in the network. GEINE3 selects the tree-based integrated learning algorithm among different model selection methods, which does not make any assumptions about the nature of gene regulation, and integrates and processes between regulation and nonlinearity. GENIE3([9](#_ENREF_9)) was used to identify human CR target genes by inputting cancer expression matrix from The Cancer Genome Atlas (TCGA)([10](#_ENREF_10)).

(IV) ARACNe([11](#_ENREF_11)) is one of the most widely used reverse-engineering algorithms in the scientific community and is extensively experimentally verified. ARACNe uses an information-theoretic framework and is a dependency measure based on reverse-engineering mutual information. The simplest model based on this approach is the "association network", which can compute mutual information between all pairs of genes and directly infer regulatory relationships between transcriptional regulatory proteins and target genes when the mutual information is greater than a given threshold. In this study, we used an updated version of ARACNe-AP([12](#_ENREF_12)), an efficient adaptive partition mutual information estimator. The downstream target gene regulatory network of human CRs was inferred by inputting TCGA expression data([10](#_ENREF_10)).

(V) TRRUST (v2) has manually proofreading the regulator-target gene relationships collected from more than 20 million life science and biomedical literature based on the text mining algorithm. We extracted the experimentally validated CR-downstream target gene pairs from TRRUST (v2)([13](#_ENREF_13)) and used them as the gold standard in this study.

Crucially, the weightiness of target genes for each CR was determined by calculating the summation of the forecasting approaches. For ensuring the high authenticity, we considered the CR-target genes supported by more than half of approaches as the genes with high reliability. Users can identify the upstream regulators of objective gene by implementing hypergeometric tests between gene(s) of interest and CR target genes with high reliability. Noticeably, the CR-target genes network was constructed by combining the CR-targets from the mentioned five methods. Users can locate and screen the subnetworks of interest based on this network, and then find the regulatory axis mediated by CRs.

**Gene set enrichment analysis**

Gene enrichment analysis is a method of analyzing gene expression information and the conclusion of it is based on a set of related genes. Thus, gene enrichment analysis increases the reliability of the research and also identifies the most relevant biological processes and biological phenomenon. Hypergeometric distribution is a discrete probability statistical distribution. It is also a commonly used method of enrichment analysis. Here, we could determine the CR that regulated genes at upstream by performing the hypergeometric tests between the genes submitted by users and highly credible CR target genes. Notably, we also could identify CRs at the upstream by executing the Hypergeometric tests between the genes of interest and experimentally confirmed target genes of CRs.

The enrichment significant *P*-value for the particular CR was calculated as：

$$P=1-\sum_{i=0}^{x-1} \frac{\left( \frac{k}{i} \right)\left( \frac{n-k}{s-i} \right)}{\left( \frac{n}{s} \right)}$$

where *n* is the number of CR target genes and *k* is the number of input genes, of which *x* genes are involved in the CR containing *s* target genes.

**Online analysis tools**

We designed and provided four analytical methods for biologists to elucidate the mechanisms of the transcriptional regulation of CR:

(I) ***CR gene set enrichment analysis***. CRs regulate genes that play important roles in diseases and make abnormal expression of these genes. To identify CRs that can regulate these abnormally expressed genes, we provided an enrichment analysis method based on the downstream regulatory network of CRs. Before the enrichment analysis, we identified the CR-downstream target genes by five methods and constructed downstream regulatory networks with high confidence. Users prepare a list of genes of interest as input to the analysis, and then select the enrichment analysis parameter (*P*-value/FDR) and the type of the background set (including experimental validation and high confidence). Based on the downstream regulatory network of CRs, CRdb performs hypergeometric tests between the given set of genes of interest and the downstream target genes of CRs to identify the upstream CRs that regulated these genes of interest (see **Gene set enrichment analysis**).

(II) ***CR binding genomic region annotation***. In this analysis, users input the genomic region of interest (in .bed format), while setting the minimum proportion value of the intersection of the input genome region and CR-binding region. CRdb calculates the intersection between the input genome region and each CR-binding region, returning information about all CRs that combine with input genomic regions with a proportion greater than the minimum proportion value (including CR name, functional type, number of peaks, the region of input peaks and the number of overlap). Furthermore, users can obtain comprehensive annotation information of overlapping genomic regions by clicking on “Details”.

(III) ***CR-TF co-occupancy analysis***. Based on the motif of TFs, users can judge the possible binding site of TFs in the CR-binding region. The FIMO software was used in this study to predict the binding location of the transcription factors on the CR ChIP-seq peak, namely, the motif enrichment. The principle of FIMO is inputting the PWM matrix of TF motif and the genomic region of CR (i.e., ChIP-seq peak region), it can predict the binding likelihood of TF by calculating the binding probability of TF motif on CR ChIP-seq peak region, thus returning the specific location with the most likely probability. We downloaded more than 3,000 motifs on DNA binding sequences from Jolma2013([14](#_ENREF_14)), JASPAR CORE 2020 vertebrates([15](#_ENREF_15)), Homeodomain([16](#_ENREF_16)), UniPROBE([17](#_ENREF_17)) and Wei2010([18](#_ENREF_18)), corresponding to the PWM matrix of 805 TFs. In the "*CR-TF co-occupancy analysis*", users can obtain the number of co-binding peaks for the CR-TF pairs by inputting multiple CRs and TFs or inputting CR(s)/TF(s). We defined the colocalization relationship between CRs and TFs by performing the intersection of CR-binding region and TF motif region. Users can find TFs that have a synergistic relationship with CRs of interest, and also CRs that cooperate with TFs inputted by themselves. By clicking on the “Details” button, users can obtain the detailed results containing Peak ID, Sample ID, Sample name, region, P-value, score and the sequence.

(IV) ***CR regulatory axis analysis***. The function of pathways is related to the development of diseases (e.g. inflammation, tumor and cancer), and research methods have been used to analyze the relationship between traditional pathways and diseases. A CR downstream pathway analysis was performed in this study to find the CRs associated with pathways. Users can submit a gene set of interest and select at least one pathway database (e. g. KEGG), while also selecting the *P*-value/FDR. First, CRdb identifies pathways significantly enriched for these genes using hypergeometric tests (see **Gene set enrichment analysis**). Next, it identifies CRs on the downstream of pathway and provides regulatory information about these CRs. Pathway ID, pathway name, CR, CR number, number of enriched genes, and *P*-value are provided in the results page. Similarly, users can get more detailed pathway information by clicking on the "Details" button. In the course of this analysis, we may uncover new regulatory patterns of not yet mentioned genes, pathways, and CRs to obtain potential new targets.

**REFERENCES**

1. Lu, J., Xu, J., Li, J., Pan, T., Bai, J., Wang, L., Jin, X., Lin, X., Zhang, Y., Li, Y. *et al.* (2018) FACER: comprehensive molecular and functional characterization of epigenetic chromatin regulators. *Nucleic Acids Res*, **46**, 10019-10033.

2. Ru, B., Sun, J., Tong, Y., Wong, C.N., Chandra, A., Tang, A.T.S., Chow, L.K.Y., Wun, W.L., Levitskaya, Z. and Zhang, J. (2018) CR2Cancer: a database for chromatin regulators in human cancer. *Nucleic Acids Res*, **46**, D918-d924.

3. Medvedeva, Y.A., Lennartsson, A., Ehsani, R., Kulakovskiy, I.V., Vorontsov, I.E., Panahandeh, P., Khimulya, G., Kasukawa, T. and Drabløs, F. (2015) EpiFactors: a comprehensive database of human epigenetic factors and complexes. *Database : the journal of biological databases and curation*, **2015**, bav067.

4. Xu, X., Bi, X., Wang, J., Gui, R., Li, T., Li, L. and Wang, B. (2022) Identification of KANSL1 as a novel pathogenic gene for developmental dysplasia of the hip. *J Mol Med (Berl)*, **100**, 1159-1168.

5. Zheng, X., Liu, Z., Zhong, J., Zhou, L., Chen, J., Zheng, L., Li, Z., Zhang, R., Pan, J., Wu, Y. *et al.* (2022) Downregulation of HINFP induces senescence-associated secretory phenotype to promote metastasis in a non-cell-autonomous manner in bladder cancer. *Oncogene*, **41**, 3587-3598.

6. Meng, L., Zhang, Y., Wu, P., Li, D., Lu, Y., Shen, P., Yang, T., Shi, G., Chen, Q., Yuan, H. *et al.* (2022) CircSTX6 promotes pancreatic ductal adenocarcinoma progression by sponging miR-449b-5p and interacting with CUL2. *Mol Cancer*, **21**, 121.

7. Wang, S., Sun, H., Ma, J., Zang, C., Wang, C., Wang, J., Tang, Q., Meyer, C.A., Zhang, Y. and Liu, X.S. (2013) Target analysis by integration of transcriptome and ChIP-seq data with BETA. *Nat Protoc*, **8**, 2502-2515.

8. Lovén, J., Hoke, H.A., Lin, C.Y., Lau, A., Orlando, D.A., Vakoc, C.R., Bradner, J.E., Lee, T.I. and Young, R.A. (2013) Selective inhibition of tumor oncogenes by disruption of super-enhancers. *Cell*, **153**, 320-334.

9. Huynh-Thu, V.A., Irrthum, A., Wehenkel, L. and Geurts, P. (2010) Inferring regulatory networks from expression data using tree-based methods. *PLoS One*, **5**.

10. Corces, M.R., Granja, J.M., Shams, S., Louie, B.H., Seoane, J.A., Zhou, W., Silva, T.C., Groeneveld, C., Wong, C.K., Cho, S.W. *et al.* (2018) The chromatin accessibility landscape of primary human cancers. *Science*, **362**.

11. Lachmann, A., Giorgi, F.M., Lopez, G. and Califano, A. (2016) ARACNe-AP: gene network reverse engineering through adaptive partitioning inference of mutual information. *Bioinformatics*, **32**, 2233-2235.

12. Rodchenkov, I., Babur, O., Luna, A., Aksoy, B.A., Wong, J.V., Fong, D., Franz, M., Siper, M.C., Cheung, M., Wrana, M. *et al.* (2020) Pathway Commons 2019 Update: integration, analysis and exploration of pathway data. *Nucleic Acids Res*, **48**, D489-d497.

13. Han, H., Cho, J.W., Lee, S., Yun, A., Kim, H., Bae, D., Yang, S., Kim, C.Y., Lee, M., Kim, E. *et al.* (2018) TRRUST v2: an expanded reference database of human and mouse transcriptional regulatory interactions. *Nucleic Acids Res*, **46**, D380-d386.

14. Jolma, A., Yan, J., Whitington, T., Toivonen, J., Nitta, K.R., Rastas, P., Morgunova, E., Enge, M., Taipale, M., Wei, G. *et al.* (2013) DNA-binding specificities of human transcription factors. *Cell*, **152**, 327-339.

15. Fornes, O., Castro-Mondragon, J.A., Khan, A., van der Lee, R., Zhang, X., Richmond, P.A., Modi, B.P., Correard, S., Gheorghe, M., Baranašić, D. *et al.* (2020) JASPAR 2020: update of the open-access database of transcription factor binding profiles. *Nucleic Acids Res*, **48**, D87-d92.

16. Berger, M.F., Badis, G., Gehrke, A.R., Talukder, S., Philippakis, A.A., Peña-Castillo, L., Alleyne, T.M., Mnaimneh, S., Botvinnik, O.B., Chan, E.T. *et al.* (2008) Variation in homeodomain DNA binding revealed by high-resolution analysis of sequence preferences. *Cell*, **133**, 1266-1276.

17. Robasky, K. and Bulyk, M.L. (2011) UniPROBE, update 2011: expanded content and search tools in the online database of protein-binding microarray data on protein-DNA interactions. *Nucleic Acids Res*, **39**, D124-128.

18. Wei, G.H., Badis, G., Berger, M.F., Kivioja, T., Palin, K., Enge, M., Bonke, M., Jolma, A., Varjosalo, M., Gehrke, A.R. *et al.* (2010) Genome-wide analysis of ETS-family DNA-binding in vitro and in vivo. *Embo j*, **29**, 2147-2160.

**SUPPLEMENTARY TABLE AND FIGURES LEGENDS**

**Supplementary Materials.** Supplementary method

**Supplementary Table S1.** The statistics of the content of CRdb.

**Supplementary Table S2.** The detailed comparison of CR associated information between CRdb and other databases.
