# Supplementary material for: CRdb: a comprehensive resource for deciphering chromatin regulators in human
Source: Nucleic Acids Res. 2022 Nov 1;51(D1):D88–D100. doi: 10.1093/nar/gkac960 (PMC9825595; doi:10.1093/nar/gkac960)
Supplement: gkac960_Supplemental_Files [file gkac960_supplemental_files.zip › Supplementary Table S1.doc]

**Supplementary Table 1.** The statistics of the content of CRdb

| **Element** | **Source** | **Number** |
| --- | --- | --- |
| Functional CR | CR2Cancer, FACER | 647 |
| CR-associated ChIP-seq sample | ENOCODE, NCBI GEO/SRA | 2,591 |
| Super-enhancer | SEdb | 1,167,518 |
| Enhancer | EnhancerAtlas, FANTOM5, ENCODE, HACER, DENdb, ENdb | 120,626,280 |
| TFBS | UCSC | 5,772,624 |
| Common SNP | dbSNP | 37,302,778 |
| Risk SNP | GWAS Catalog, GWASdb (v2) | 351,728 |
| eQTL | SCAN, seeQTL, PancanQTL, Oncobase | 2,886,133 |
| Accessible chromatin | ENCODE, NCBI GEO/SRA | 199,881,570 |
| 450K | ENCODE | 32,099,123 |
| WGBS | ENCODE | 176,535,821 |
| Chromatin interaction | 3D Genome Browser, 4DGenome, OncoBase | 34,414,944 |
