# Supplementary material for: CRdb: a comprehensive resource for deciphering chromatin regulators in human
Source: Nucleic Acids Res. 2022 Nov 1;51(D1):D88–D100. doi: 10.1093/nar/gkac960 (PMC9825595; doi:10.1093/nar/gkac960)
Supplement: gkac960_Supplemental_Files [file gkac960_supplemental_files.zip › Supplementary Table S2.doc]

**Supplementary Table 2.** The detailed comparison of CR associated information between CRdb and other databases

| **Attribution** | **Data type/Specific function** | **CRdb** | **CR Cistrome** | **FACER** | **CR2Cancer** |
| --- | --- | --- | --- | --- | --- |
| Functional CR number | Number of human functional CR | 647 | 49 | 519 | 429 |
| CR ChIP-seq sample number | Number of human samples | 2,591 | 1,566 | － | 1,359 |
| Quality control | Reads (Number of sample reads within analysed chromosomes) | √ | √ | － | － |
|  | Dup% (Percentage of MapQ filter passing reads marked as duplicates) | √ | － | － | － |
|  | ReadL | √ | － | － | － |
|  | FragLen (estimated fragment length by cross-coverage method) | √ | － | － | － |
|  | RelativeCC (Cross-Coverage score at the fragment length over Cross-coverage at the read length) | √ | － | － | － |
|  | SSD (SSD score htSeqTools) | √ | － | － | － |
|  | RIP% (Percentage of reads within peaks) | √ | √ | － | － |
| Peak annotation visualization | Genomic feature distribution | √ | － | － | － |
| Upstream annotation | Promoter | √ | － | － | － |
|  | Enhancer | √ | － | － | － |
|  | Super-enhancer | √ | － | － | － |
|  | MiRNA | √ | √ | √ | √ |
| Region annotation | Enhancer | √ | － | － | － |
|  | Super-Enhancer | √ | － | － | － |
|  | Accessible Chromatin | √ | － | － | － |
|  | Chromatin Interaction | √ | － | － | － |
|  | TAD | √ | － | － | － |
|  | Common SNP | √ | － | √ | － |
|  | LD SNP | √ | － | √ | － |
|  | risk SNP | √ | － | √ | － |
|  | eQTL | √ | － | － | － |
|  | CRISPR/Cas9 target site | √ | － | － | － |
|  | Methylation site | √ | － | √ | √ |
| Functional annotation | GO Term | √ | － | － | √ |
|  | Cancer Hallmark | √ | － | √ | － |
|  | Survival | √ | － | √ | √ |
|  | PPI | √ | － | √ | √ |
|  | Pathway sources | 10^a^ | － | － | 2^b^ |
|  | Expression sources | TCGA, CCLE, GTEx, ENCODE | － | TCGA | TCGA, CCLE, HPA |
|  | Disease information sources | DisGeNET, GAD | √ | TCGA | √ |
| Downstream target genes | Strategies of CR associated genes | BETA, Genemapper, GENIE3, ARACNe and TRRUST | － | － | ARACNe, BETA |
| Online analysis | CR gene set enrichment | √ | － | － | － |
|  | CR binding genomic region annotation | √ | － | － | － |
|  | CR-TF co-occupancy analysis | √ | － | － | － |
|  | CR regulatory axis analysis | √ | － | － | － |

^a^Pathway were collected from ten resources: KEGG, Reactome, PANTHER, SMPDB, NetPath, PID, HumanCyc, CTD, WikiPathways, INOHstarBase v2.0 and EuRBPDB.

^b^Pathway were obtained from two resources: KEGG, Reactome.
